# Ruptured endometrioma: main imaging findings

**DOI:** 10.1590/0100-3984.2017.0092

**Published:** 2018

**Authors:** Eduardo Kaiser U. N. Fonseca, Bruna Bringel Bastos, Fernando Ide Yamauchi, Ronaldo Hueb Baroni

**Affiliations:** 1 Hospital Israelita Albert Einstein, São Paulo, SP, Brazil.

**Figure 1:**
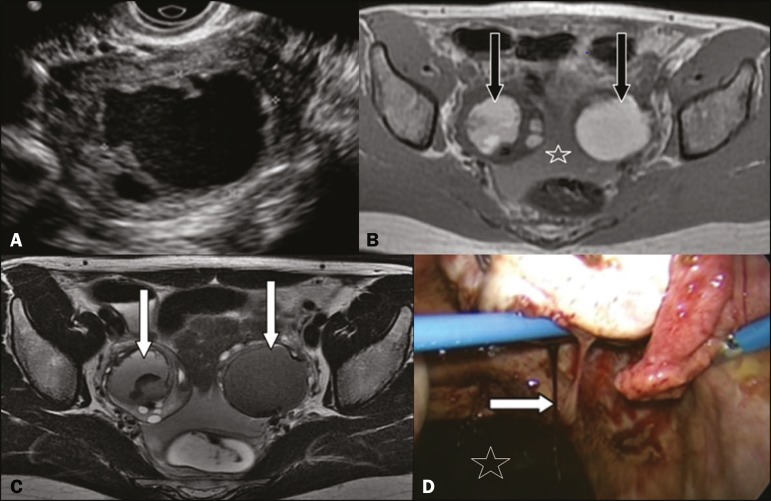
TVUS showing a cyst with irregular contours and hypoechoic content in the right ovary. **B,C:** MRI of the pelvis showing formations with high signal intensity in T1-weighted images (**B**) and low signal intensity in T2-weighted images (“shading” in **C**, arrow) in both ovaries, with irregular contours in the right ovary. Fluid content in the pelvic cavity with high signal intensity on the T1-weighted image (**B**), indicating hemoperitoneum. This set of findings is suggestive of bilateral endometriomas, with signs of rupture of the right endometrioma. **D:** Image obtained in laparoscopic access of the pelvic cavity showing active bleeding (arrow) in the right ovary and blood content collected in the pelvic recess (star), findings that correspond to those seen on TVUS and MRI.

Dear Editor,

A 28-year-old woman presented with a 12-h history of acute pelvic pain. Physical examination revealed signs of peritonitis, and laboratory tests showed mild anemia. The patient underwent transvaginal ultrasound (TVUS) of the pelvis (Figure 1A) and magnetic resonance imaging (MRI) of the pelvis (Figures 1B and 1C). She also underwent laparoscopy, which confirmed the presence of bilateral ovarian endometriosis, with a rupture on the right side (Figure 1D).

The presumptive diagnosis of endometriosis is based on a clinical history consistent with the diagnosis and abnormal laboratory tests, including elevation of CA-125, a marker that, although nonspecific, is usually elevated in women with the disease^(^^1^^)^. Despite the important role of imaging examinations, notably TVUS and MRI of the pelvis in the diagnosis and staging of endometriosis, it should be noted that the gold standard for the definitive diagnosis is still laparoscopy^(^^1^^,^^2^^)^.

The ovaries are among the sites most commonly affected by endometriosis (in 20-40% of cases). Endometriomas are thick-walled cysts containing dark, thick degenerated blood products. In some cases, there can be a fluid-fluid level, representing bleeding of various chronologies, giving them the typical macroscopic appearance of “chocolate cysts”, which can be transposed to imaging exams^(^^3^^)^. They are bilateral in approximately 50% of cases^(^^2^^,^^4^^)^.

The rupture of an endometrioma is a rare event, with an estimated incidence of less than 3% among women of childbearing age who are known to have endometriomas^(^^5^^)^. This situation occurs more commonly during pregnancy, due to hormonal stimulation of endometrial stromal elements^(^^2^^)^, albeit with larger (≥ 6.0 cm) lesions^(^^6^^)^.

The imaging aspect of endometrioma is that of an ovarian cyst with heterogeneous content, irregular contours, and parietal discontinuity, together with hemoperitoneum, which can be seen as heterogeneous fluid content on ultrasound and as a collection with a hyperintense signal in T1-weighted MRI sequences. In an emergency setting, its presentation may mimic other acute gynecological conditions, such as corpus luteum, ectopic gestation, and even spontaneous hemoperitoneum^(^^7^^,^^8^^)^. In addition, the rupture of endometriomas can significantly increase serum CA-125 levels, mimicking ovarian epithelial neoplasms^(^^9^^)^. However, a history of endometrioma, previous examinations demonstrating endometriomas, or endometriomas accompanied by peritoneal blood content in emergency imaging studies should raise the suspicion of spontaneous rupture.

The importance of the preoperative diagnosis is to support treatment strategies. Although some milder cases can be managed conservatively, there is a tendency toward greater use of early surgical exploration because of long-term undesirable effects of cyst fluid in the peritoneal cavity, such as adhesions, pelvic pain, and infertility^(^^6^^)^. In addition, the presumptive diagnosis of ruptured endometrioma, rather than ovarian neoplasms, facilitates the decision to perform laparoscopic exploration and allows the surgeon to perform the procedure with greater confidence.
